# Effects of Two-Phase Treatment with Functional Appliances Followed by Extraction versus One-Phase Treatment with Extraction in Class II Growing Patients: A Case–Control Study

**DOI:** 10.3390/jcm11247428

**Published:** 2022-12-15

**Authors:** Ka Fai Wong, Wener Chen, Jianhan Ren, Yanqi Yang, Yifan Lin

**Affiliations:** Division of Paediatric Dentistry and Orthodontics, Faculty of Dentistry, The University of Hong Kong, Hong Kong SAR, China

**Keywords:** Class II skeletal relationship, two-phase treatment, functional appliance, extraction

## Abstract

Objectives: Fixed appliance treatment with premolar extraction is often required after functional appliance treatment to relieve crowding and improve facial aesthetics in the Asian population. This study compared the treatment efficacy of two approaches for treating Class II division 1 malocclusion: functional appliance followed by fixed appliance treatment with extraction (two-phase) and fixed appliance treatment with extraction (one-phase). Methods: Growing skeletal Class II patients with an overjet of ≥6 mm treated with two- or one-phase orthodontics were included. The two groups consisted of 29 patients (mean age = 12.55) and 30 patients (mean age = 12.72), respectively. Pre- and post-treatment cephalograms were analysed and skeletal, dental, and soft tissue characteristics were compared using independent *t*-tests. Treatment changes were compared within and between groups using paired and independent *t*-tests, respectively. Stepwise discriminant analysis was performed to identify the variables that best predicted pre-treatment group allocations. Results: At baseline, there were no significant between-group differences in age, gender, cervical vertebral maturation, or overjet. The two-phase group had greater Class II skeletal discrepancies (ANB angle and Wits appraisal). During treatment, the two-phase group showed greater improvements in intermaxillary relationship and facial convexity compared with the one-phase group (*p* < 0.01). Following treatment, the two-phase group had a greater L1/APog distance (*p* < 0.05). Facial convexity and Wits appraisal were identified as parameters significantly influencing the clinicians’ decision to use a one- or two-phase approach. Conclusions: In patients requiring premolar extraction, two-phase (vs. one-phase) treatment produced greater improvements in the intermaxillary relationship and facial convexity.

## 1. Introduction

Class II malocclusion is the second most prevalent malocclusion, accounting for 20.5% of the Chinese population [1,2]. Growth modification with a functional appliance is common as a first-phase treatment modality for Class II patients with mandibular retrognathism [3,4]. Second-phase treatment with fixed appliances frequently follows to correct dental crowding, adjust teeth alignment, and improve occlusion [4].

However, the effects of functional appliances on Class II patients are controversial. Many studies have suggested that the treatment effects of functional appliances are mainly dentoalveolar, with minimal skeletal effect [5,6]. Moreover, the effects of growth modification with functional appliances might not be maintained in the long term [7].

Camouflage orthodontic treatment, which usually incorporates premolar extractions, is another treatment option for dental compensation in Class II malocclusion correction [8]. By extracting two or four premolars, teeth can be aligned, the overjet can be normalised, and proclined incisors can be corrected.

Marked ethnic differences in soft tissue characteristics between Asian and Caucasian populations are well established. In general, the Chinese population presents with a more acute nasolabial angle, more protrusive upper and lower lips, and a more convex facial profile, accounting for the higher extraction rate compared with the Caucasian population [9,10,11].

For Class II patients with crowding and/or a protrusive profile, orthodontists often recommend functional appliances followed by premolar extraction to relieve crowding and/or improve the facial profile [12,13]. In other words, patients may still require extractions during the second phase of treatment after a first-phase functional appliance treatment. Therefore, when treating such patients, a question remains unanswered: do functional appliances produce additional benefits for patients who need extractions during fixed appliance treatment? Whether a single-phase fixed appliance can produce treatment effects equivalent to those achieved with two-phase treatment remains unknown.

The current study aims to compare the skeletal, dentoalveolar, and soft tissue effects between patients with Class II division 1 malocclusion treated with functional appliances followed by fixed appliances with premolar extraction (two-phase) and those directly treated with fixed appliances with premolar extraction (one-phase).

## 2. Materials and Methods

This study was approved by the Institutional Review Board of the University of Hong Kong/Hospital Authority Hong Kong West Cluster (Reference number: UW 22-501).

### 2.1. Sample Size Calculation

A minimum of 28 patients per group were required to detect a clinically significant difference of 3° in facial convexity (G’-Sn-Pog’) between the two groups based on the previously reported standard deviations of 3.9° (power = 0.8) [14].

### 2.2. Sample Selection

This was a retrospective case–control study. The sample comprised 59 skeletal Class II patients from the Prince Philip Dental Hospital, the University of Hong Kong. The inclusion criteria were as follows: (1) an age between 9 and 16 years at the start of treatment, (2) skeletal Class II with an ANB angle ≥4°, (3) Class II division 1 malocclusion with an initial overjet ≥6 mm, and (4) treatment with either a functional appliance followed by fixed appliances with extraction (two-phase) or fixed appliances with extraction alone (one-phase). The exclusion criteria were hypodontia, cleft lip and palate, or other craniofacial abnormalities.

The two-phase group consisted of 29 patients (13 males and 16 females) treated with Herbst appliances (18 patients) or Twin-block appliances (11 patients), followed by a second phase of treatment with fixed appliances and premolar extractions. The cephalograms were examined at baseline (T1, mean age = 12.55 years), at the end of the functional appliance (T2, mean age = 13.80 years), and at the end of the fixed appliances (T3, mean age = 17.37 years). The one-phase group consisted of 30 patients (13 males and 17 females) treated with fixed appliances with premolar extractions. The cephalograms were examined at baseline (T1, mean age = 12.72 years) and at the end of the fixed appliances (T2, mean age = 15.90 years). All included patients had four premolar extractions during treatment.

### 2.3. Cephalometric Analysis

**Figure 1:**
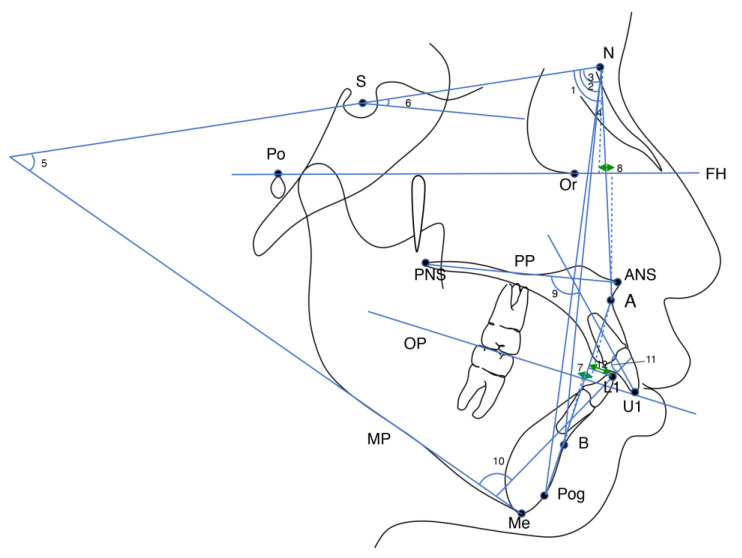
Cephalometric landmarks and skeletal and dental measurements: S, Sella; N, Nasion; Po, Porion; Or, Orbitale; ANS, anterior nasal spine; PNS, posterior nasal spine; A, Subspinale; B, Supramentale; Pog, Pogonion; Me, Menton; U1, tip of maxillary central incisor; L1, tip of mandibular central incisor; FH, Frankfort horizontal plane; PP, palatal plane (ANS-PNS); OP, occlusal plane; MP, mandibular plane.

**Figure 2:**
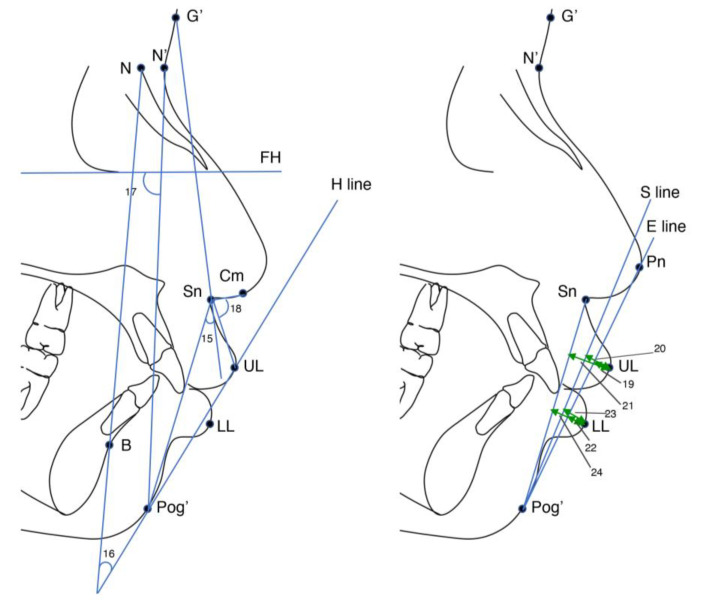
Cephalometric landmarks and soft tissue measurement. G’, soft tissue Glabella; N’, soft tissue Nasion; Pn, Pronasale; Cm, columella; Sn, Subnasale; UL, upper lip anterior; LL, lower lip anterior; Pog’, soft tissue Pogonion; H line, UL-Pog’; E line, Pn-Pog’; S line, middle of the S-shaped curve of nasal-Pog’.

All lateral cephalograms were taken in the natural head position with the lips at rest and the teeth positioned in centric occlusion. The cephalograms were digitised using Dolphin Imaging Software version 11.5 (Dolphin Imaging and Management Solutions, Patterson Dental Supply, Inc., Chatsworth, CA, USA). Twenty landmarks were defined on each cephalogram, and eight skeletal, six dental, and ten soft tissue variables were measured (Figure 1 and Figure 2). The definitions of skeletal, dental, and soft tissue measurements are shown in Table 1.

### 2.4. Method Error Study

To assess the method error, 10 cephalograms were randomly selected from the groups to be redigitised 2 weeks later by the same investigator. The errors were calculated using Dahlberg’s formula, *d* = √(Σ*d*^2^/2*n*), where *d* is the difference between the first and second measurements, and *n* is the sample size that was remeasured. The errors for the linear and angular measurements were within 0.85 mm and 0.96°, respectively.

### 2.5. Statistical Analysis

The normality of the data was tested using Shapiro–Wilk tests. Pre- and post-treatment skeletal, dental, and soft tissue characteristics were compared between the two groups using independent *t*-tests. Treatment changes were compared within the groups using paired *t*-tests and between the groups using independent *t*-tests. Stepwise discriminant analysis was performed to characterise the anatomic basis of the initial treatment decision. The level of significance was set at 0.05. Statistical analyses were conducted using SPSS software (version 26; IBM, Armonk, NY, USA).

## 3. Results

The demographic data of the two-phase group and one-phase group are shown in Table 2. No significant differences were detected between the two groups in terms of gender distribution (*p* = 0.908), age (*p* = 0.664), and cervical vertebral maturation stage at T1 (*p* = 0.795). The total treatment duration was significantly longer in the two-phase group (average = 4.82 ± 1.58 years) than in the one-phase group (average = 3.18 ± 0.91 years, *p* < 0.001). The pre-treatment characteristics, within-group and between-group treatment changes, and post-treatment characteristics are shown in Table 3, Table 4 and Table 5 respectively.

### 3.1. Pre-Treatment Cephalometric Characteristics

At baseline (T1), no significant difference in overjet was found between the two groups (*p* = 0.243). However, the two-phase group had a greater Class II skeletal discrepancy than the one-phase group, shown by a significantly greater ANB angle (*p* = 0.017) and Wits appraisal (*p* = 0.005). The two-phase group also had a more convex soft tissue profile, with a significantly greater facial convexity (*p* = 0.002) and Holdaway angle (*p* = 0.045).

### 3.2. Skeletal Changes

For the two-phase group, significant decreases in SNA, ANB, and Wits appraisal and significant increases in SNB and SNPog were detected in the first phase (T1–T2; SNA: *p* = 0.011, other variables: *p* < 0.001). The significant skeletal changes over the entire treatment period (T1–T3) were similar to those observed in the first phase (T1–T2), except that SNB was not significantly different at T3 (*p* > 0.05). Overall, significant reductions in SNA (*p* = 0.002), ANB (*p* < 0.001), Wits appraisal (*p* = 0.001), and Naperp-A (*p* = 0.011) and a significant increase in SNPog (*p* = 0.020) occurred at T3 compared with T1. For the one-phase group, a significant decrease in the ANB angle (*p* = 0.001) and a significant increase in SNPog angle (*p* = 0.008) were observed.

Comparing the overall treatment changes between the two groups, the reduction in ANB in the two-phase group was significantly larger than that in the one-phase group by 1.19° (*p* = 0.001). Significant decreases in Wits appraisal (*p* = 0.002) and Naperp-A (*p* = 0.033) occurred in the two-phase group. At the end of treatment, no significant differences in any of the skeletal parameters between the two groups were detected (*p* > 0.05).

### 3.3. Dental Changes

In the two-phase group, overjet, overbite, and U1/MxPl significantly decreased while L1/MnPl and L1/APog significantly increased from T1 to T2 (all *p* < 0.01). Overall, the significant dental changes across the treatment period (T1–T3) were decreases in overjet, overbite, U1/MxPl, and L1/MnPl and an increase in U1/L1 (all *p* < 0.01). However, no significant difference was detected regarding L1/APog distance during T1-T3 (*p* > 0.05).

In the one-phase group, significant decreases in overjet (*p* < 0.001), overbite (*p* < 0.001), U1/MxPl (*p* < 0.001), L1/MnPl (*p* = 0.007), and L1/APog (*p* = 0.010) and a significant increase in U1/L1 (*p* < 0.001) were found after treatment. Intergroup analysis showed no significant differences in overall dental changes between the two groups (*p* > 0.05). After treatment, the only significant between-group difference was L1/APog, which was 1.21 mm greater in the two-phase group than the one-phase group (*p* = 0.022).

### 3.4. Soft Tissue Changes

In the two-phase group, significant decreases in upper lip protrusion (UL-E, UL-S, and UL protrusion: all *p* < 0.001), Holdaway angle (*p* < 0.001), and facial convexity (G’-Sn-Pog’: *p* = 0.001) and significant increases in the facial angle (FH-N’Pog’: *p* = 0.028) and nasolabial angle (*p* = 0.031) occurred from T1 to T2. Overall, the significant soft tissue changes across the treatment period (T1–T3) were decreases in upper (UL-E, UL-S, and UL-SnPog’: all *p* < 0.001) and lower lip protrusion (LL-E, LL-S, and LL-SnPog’: all *p* < 0.001), Holdaway angle (*p* < 0.001), and facial convexity (G’-Sn-Pog’: *p* = 0.001) and a significant increase in the nasolabial angle (*p* = 0.035).

In the one-phase group, significant decreases in facial convexity (*p* = 0.003), upper (all *p* < 0.001) and lower lip protrusion (all *p* < 0.001), and Holdaway angle (*p* < 0.001) and a significant increase in the facial angle (*p* = 0.042) were noted. Notably, intergroup analysis showed that the two-phase group had a 2.23° greater reduction in facial convexity than in the one-phase group (*p* = 0.021). After treatment, there were no significant differences in any soft tissue parameters between the two groups (*p* > 0.05).

### 3.5. Discriminant Analysis

Stepwise discriminant analysis was performed to identify the variables that best predict the pre-treatment group allocations. Two variables were significantly incorporated into the discriminant function (*p* < 0.001): facial convexity and Wits. The equation for the individual scores was as follows: Individual score = −2.173 + 0.223 * (Wits) + 0.160 * (facial convexity).

**Figure 3:**
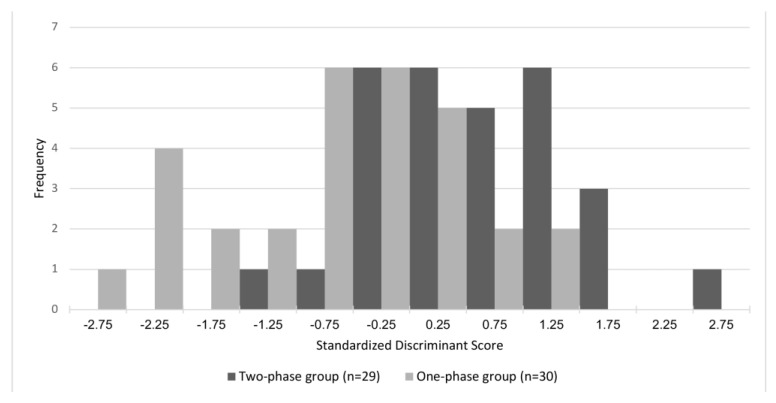
Standardised discriminant scores of the two-phase and one-phase group. Patients in the two-phase treatment group tended to have more positive discriminant scores, whereas patients in the one-phase treatment group tended to have more negative discriminant scores.

The critical score was −0.008, which was the mean centroid of the two groups. For patients with individual scores greater than −0.008, clinicians tended to select the two-phase treatment, whereas they tended to select one-phase treatment for patients with individual scores less than −0.008. In this model, 71.2% of the original grouped cases were correctly classified (Table 6). Figure 3 shows the discriminant scores in the two-phase and one-phase groups.

## 4. Discussion

Most previous studies on treatment with a functional appliance followed by a fixed appliance have investigated cases without extractions [15,16]. However, extractions are very common during fixed appliance treatment in Asian populations due to the amount of crowding and protrusion of the facial profile [2,17]. Even after functional appliance treatment, extractions are often required to alleviate crowding and improve the facial profile and lip competency [12,13,18]. Therefore, we aimed to determine whether functional appliances produce benefits for patients who need premolar extractions during fixed appliance treatment and whether a one-phase fixed appliance treatment approach can produce benefits equivalent to those achieved with two-phase treatment.

### 4.1. Skeletal Changes

In the short term, the functional appliance produced favourable skeletal responses, including improvement of the intermaxillary relationship, inhibition of maxillary growth, and enhancement of mandibular growth in the two-phase group, consistent with previous studies [19,20,21].

The long-term effects of functional appliance treatment on mandibular growth are debated. A study on Herbst appliance treatment reported only a temporary inhibition of maxillary growth, a temporary increase in mandibular growth, and improvement in the intermaxillary relationship during active treatment; however, the treatment changes were reversed during the post-treatment period [22]. In the current study, the two-phase group showed a significant increase in the SNB angle from T1 to T2; however, no significant difference in SNB was observed at T3 compared with T1. Our results are consistent with the idea that functional appliances take a mortgage on mandibular growth [23]. Functional appliances prevent mesial movement of the maxillary dentition and utilise the normal pattern of facial growth, rather than mandibular growth modification, to achieve Class II correction [24]. Additionally, retraction of the lower incisors after lower premolar extraction during T2–T3 could result in the backward remodelling of point B compared with that at the end of the first phase (T2), which could explain the unchanged SNB angle at T3. It is worth mentioning that SNPog significantly increased from T1 to T3, which implies bone remodelling or forward positioning of the chin.

The one-phase group had significantly reduced ANB and SNPog angles after treatment, which implies a relative improvement in the anteroposterior relationship. Regarding treatment changes between the two groups, the decreases in the SNA angle, ANB angle, Wits appraisal, and Naperp-A were more significant in the two-phase group than in the one-phase group. Interestingly, no significant differences in skeletal parameters were found between the two groups at the end of treatment, despite the greater ANB angle and Wits appraisal of the two-phase group before treatment. The results indicate that although the two-phase group had a more severe anteroposterior discrepancy before treatment, they caught up with the one-phase group after two phases of treatment.

### 4.2. Dental Changes

Significant dental improvements were observed in both groups, and no significant between-group difference in the overall treatment changes was detected. At the end of the treatment, the only significantly different parameter was the L1/APog distance, which was greater in the two-phase group than in the one-phase group. In the two-phase group, the lower incisors were significantly proclined during T1–T2, resulting in an increase in the L1/APog distance of 3.10 mm and in L1/MnPl of 5.98°. Such changes may be attributable to the mesially directed force of the functional appliance on the mandibular dentition [16]. In the T2–T3 phase, the lower incisors retracted, leading to a reduction in the L1/APog distance of 3.55 mm and in L1/MnPl of 9.69°. Such round-tripping of the lower incisors may not be observed in two-phase non-extraction treatment but is rather common in two-phase extraction cases [12,18]. Round-tripping increases the treatment time as well as the associated risks of root resorption, periodontal damage, white spot lesions, and costs [25,26]. By contrast, one-phase fixed appliance treatment avoids unnecessary round-tripping and produced similar dental compensations.

### 4.3. Soft Tissue Changes

Generally, the Chinese population presents with a more protrusive profile, more acute nasolabial angle, and more protrusive upper and lower lips than the Caucasian population [9,10,11]. By advancing the mandible, a functional appliance may not fully correct the Class II profile but rather transform it into a profile of bimaxillary dentoalveolar protrusion [12]. Furthermore, in a study in which Chinese and White judges rated the lip position on profile images, both Chinese dentists and laypeople preferred a more retrusive profile and were more likely to rate protrusive profiles as unattractive [27]. Therefore, in some cases, the crowded dentition, deep curve of Spee, proclined incisors, and convex profile led to the decision of premolar extraction after functional appliances.

At T1, the facial convexity (G’-Sn-Pog’) of the two-phase group was larger, indicating a more convex profile than that of the one-phase group. During treatment, the soft tissue profile significantly improved in both groups, as shown by the increase in the nasolabial angle and decreases in the facial convexity angle and upper and lower lip protrusion. To some extent, the functional appliance may improve the intermaxillary relationship and reduce mentalis muscle tension, resulting in improved facial convexity and chin aesthetics in the two-phase group [28]. However, the significant reductions in upper and lower lip protrusion in both groups were attributable to the premolar extractions followed by upper and lower incisor retraction. Regarding the soft tissue changes between the two groups, the reduction in facial convexity was greater in the two-phase group than in the one-phase group (difference = 2.23°), explaining the similar facial convexity in the two groups at the end of the treatment. The two groups presented with similar profiles and exhibited no significant differences in soft tissue parameters after treatment.

### 4.4. Discriminant Analysis

Discriminant analysis was conducted to identify the variables that might affect clinicians’ decisions to select one or two-phase treatment for Class II malocclusion. Livieratos and Johnston assessed the dental models and cephalometric variables that may predict which treatment option a clinician will choose for Class II malocclusion [23]. In their study, age, overjet, and molar relationship were identified, indicating that there was a tendency for younger patients with more severe malocclusions to be treated using a two-phase approach. In the current study, patients were matched according to gender, age, and skeletal maturation; thus, only cephalometric variables were included to generate the discriminant function. As a result, facial convexity and Wits appraisal were identified as clinical parameters that significantly affect the clinician’s decision for group allocation: patients with greater facial convexity and Wits appraisal tended to be treated via two-phase treatment. In this way, more than 70% of the patients were correctly classified. However, it is likely that the treatment decisions were not so clear-cut in practice; as a result, there was enough noise in the system to produce considerable overlap between groups.

### 4.5. Type of Functional Appliances

The two-phase group consisted of subjects treated with the Herbst (n = 18) or Twin-block (n = 11) appliance, which might be a confounding factor in the two-phase group. However, previous studies have found that the overall effects and general modes of action are similar among different functional appliances [29,30,31]. For example, the randomised controlled trial by O’Brien and colleagues compared the treatment effects of the Herbst and Twin-block appliances for Class II malocclusion and found no significant difference between groups regarding the skeletal and dental changes [29]. Baysal and Uysal concluded that the effects of Herbst and Twin-block treatment on the soft tissue profile were similar; the appliances both significantly improved the soft tissue profile [30]. Furthermore, a systematic review by Pacha et al. comparing the effectiveness of fixed and removable functional appliances concluded that there was a lack of data relating to patient experiences and potential complications during Class II treatment [31]. Our subgroup analysis also found no difference in the treatment changes between patients using Herbst and Twin-block appliances (Supplementary Table S1). Therefore, functional appliances were pooled together aiming to compare the differences between two-phase and one-phase treatment changes.

### 4.6. Treatment Duration

Two-phase treatment involving a functional appliance followed by fixed appliances usually takes longer than a one-phase fixed appliance approach [32,33]. In the current study, the treatment duration for two-phase treatment (average = 4.82 ± 1.58 years) was significantly longer than for one-phase treatment (average = 3.18 ± 0.91 years). However, the duration of fixed appliance treatment was similar in the two groups. This result contradicts the findings of some studies that suggest that a first-phase functional appliance treatment shortens the duration of the second-phase fixed appliance treatment [34,35]. The underlying reason might be that previous studies adopted non-extraction treatment plans during the second phase, whereas all patients in the current study underwent extractions. The treatment method chosen (extraction vs. non-extraction) has been reported to influence the treatment time; extractions are linked to longer treatment times, although the conclusions remain controversial [36,37]. Malocclusion characteristics have also been suggested to influence treatment time. Class II malocclusion predicted a longer treatment duration than Class I [36,38]. In the current study, patients in the two-phase group achieved Class I malocclusion after functional appliance therapy; however, such improvements did not reduce the second-phase fixed appliance treatment time. The similarity in fixed appliance treatment duration between the groups might be explained by other factors, such as patient cooperation, the university setting, and the stability of functional appliance treatment. Nevertheless, the difficulty of second-phase fixed appliance treatment might be less than that of one-phase fixed appliance treatment. For one-phase treatment, the upper incisors need to be significantly retracted to correct the overjet, and lower molars need to be mesialised to achieve a Class I molar relationship. During these processes, anchorage control may be demanding. Using a functional appliance first to correct the intermaxillary relationship may improve the manageability of the remaining malocclusion.

### 4.7. Limitations

The limitations of this study were the lack of an untreated control group and the retrospective design. A contemporary untreated control sample should have been used to compare the skeletal, dentoalveolar, and soft tissue changes during growth; however, it would be unethical to leave a Class II patient untreated during the optimal treatment window. A prospective randomised controlled trial will provide parallel pre-treatment groups and prevent bias in patient assignment. In addition, although most patients had passed their pubertal growth spurt by the end of treatment, it would be beneficial to continue patient follow-up to compare the stability of the two treatment approaches.

## 5. Conclusions

The major findings are summarised as follows:

1. Patients with greater facial convexity and Wits appraisal tended to be treated using a two-phase approach.
2. Compared with the one-phase group, the two-phase group had a greater skeletal Class II discrepancy and more convex profiles before treatment. At the end of treatment, the two groups presented with similar skeletal, dental, and soft tissue profiles, except that the two-phase group had greater L1/APog distance.
3. During treatment, the two-phase group exhibited greater improvements in the intermaxillary relationship and facial convexity than the one-phase group.
4. The duration of two-phase treatment was significantly longer than that of one-phase treatment. Functional appliance treatment may not reduce the second-phase fixed appliance treatment duration if premolar extractions are performed.

## Figures and Tables

**Table 1:** Definitions of skeletal, dental, and soft tissue measurements.

Skeletal Measurement	Definitions
1. SNA (^o^)	Anteroposterior position of the maxilla relative to the cranial base
2. SNB (^o^)	Anteroposterior position of the mandible relative to the cranial base
3. ANB (^o^)	Sagittal relationship of maxillary base and mandibular base
4. SNPog (^o^)	Protrusion of the bony chin relative to the cranial base
5. SN/MnPl (^o^)	Mandibular plane angle
6. SN/MxPl (^o^)	Maxillary plane angle
7. Wits (mm)	Sagittal relationship of anterior maxillary and mandibular (distance between perpendiculars from points A and B on functional occlusal plane)
8. Naperp-A (mm)	Maxillary protrusion (distance from Nasion perpendicular line to point A)
Dental measurement	
9. U1/MxPl (^o^)	Inclination of upper incisor
10. L1/MnPl (^o^)	Inclination of lower incisor
11. U1/L1 (^o^)	Inter-incisal angle
12. L1/APog (mm)	Protrusion of lower incisor
13. Overjet (mm)	Distance between incisal edges of maxillary and mandibular central incisors, which is parallel to occlusal plane
14. Overbite (mm)	Distance between incisal edges of upper and lower central incisors, perpendicular to occlusal plane
Soft tissue measurement	
15. Facial Convexity (G’-Sn-Pog’) (^o^)	Convexity of the soft tissue profile
16. Holdaway Angle (N’Pog’ to H line) (^o^)	Prominence of upper lip in relation to the overall soft tissue profile. Formed by NB line (Nasion to point B) and the H line (line tangent to upper lip and soft tissue pogonion)
17. Facial Angle (FH-N’Pog’) (^o^)	Prominence of soft tissue chin
18. Nasolabial angle (Cm-Sn-UL) (^o^)	Prominence of upper lip in relation to nasal base
19. UL-E (mm)	Prominence of upper lip in relation to E line
20. UL-S (mm)	Prominence of upper lip in relation to S line
21. UL Protrusion (UL-SnPog’) (mm)	Protrusion of upper lip in relation to SnPog’ line
22. LL-E (mm)	Prominence of lower lip in relation to E line
23. LL-S (mm)	Prominence of lower lip in relation to S line
24. LL Protrusion (LL-SnPog’) (mm)	Protrusion of lower lip in relation to SnPog’ line

**Table 2:** Demographic characteristics of the study subjects.

	Two-Phase (n = 29)	One-Phase (n = 30)	*p*-Value
Mean age (years)	12.55	12.72	0.664 ^#^
Treatment time (years)	4.82 ± 1.58	3.18 ± 0.91	<0.001 ^#^
Gender			0.908 ^##^
Male	13	13	
Female	16	17	
Cervical vertebral maturation (CVM) stage			0.795 ^##^
CVM Stage 2	1	1	
CVM Stage 3	13	11	
CVM Stage 4	15	18	

^#^, Independent *t*-test; ^##^, Chi-square test.

**Table 3:** Comparison of pre-treatment cephalometric variables.

	Two-Phase Group (n = 29)		One-Phase Group (n = 30)		*p* Value ^#^
Variables	Mean	SD	Mean	SD	
Skeletal					
SNA (^o^)	82.34	3.11	81.00	3.03	0.099
SNB (^o^)	76.29	2.76	75.96	2.92	0.823
ANB (^o^)	6.04	1.41	5.04	1.72	0.017 ^*^
SNPog (^o^)	76.22	3.04	76.20	3.20	0.973
SN/MnPl (^o^)	36.48	5.39	36.87	6.44	0.803
SN/MxPl (^o^)	9.17	2.72	10.14	3.23	0.220
Wits (mm)	−0.67	2.68	−2.78	2.93	0.005 *
Naperp-A (mm)	−0.07	3.08	−1.48	3.34	0.097
Dental					
U1/MxPl (^o^)	122.70	6.40	121.43	6.18	0.442
L1/MnPl (^o^)	100.44	5.57	98.60	7.66	0.295
U1/L1 (^o^)	109.56	8.14	113.23	8.25	0.091
L1/APog (mm)	4.43	2.24	4.20	2.51	0.703
Overjet (mm)	7.67	2.21	6.93	2.60	0.243
Overbite (mm)	3.29	1.70	3.36	1.84	0.879
Soft tissue					
Facial Convexity (^o^)	18.27	4.07	15.05	3.69	0.002 *
Holdaway Angle (^o^)	19.95	3.78	17.78	4.34	0.045 *
Nasolabial angle (^o^)	89.01	11.72	92.12	10.08	0.277
Facial angle (^o^)	87.52	2.76	87.40	2.88	0.877
UL-E (mm)	2.33	2.23	1.84	2.40	0.419
UL-S (mm)	4.86	1.91	4.58	2.04	0.581
UL-SnPog’ (mm)	7.93	1.85	8.00	1.79	0.885
LL-E (mm)	4.35	2.60	3.74	2.33	0.348
LL-S (mm)	5.29	2.56	5.16	2.14	0.837
LL-SnPog’ (mm)	6.57	2.56	6.77	2.04	0.744

^#^ Independent *t*-test; * *p* < 0.05.

**Table 4:** Intragroup and intergroup comparison of treatment changes.

	Two-Phase (n = 29)						One-Phase (n = 30)			
	T1-T2			T1-T3			T1-T2			
Variables	Mean	SD	*p* Value ^#^	Mean	SD	*p* Value ^#^	Mean	SD	*p* Value ^#^	*p* Value ^##^
Skeletal										
SNA (^o^)	−0.66	1.31	0.011 *	−1.39	2.16	0.002 *	−0.40	1.92	0.264	0.067
SNB (^o^)	0.99	1.31	<0.001 *	0.66	2.47	0.163	0.44	1.58	0.135	0.691
ANB (^o^)	−1.63	1.13	<0.001 *	−2.04	1.36	<0.001 *	−0.85	1.25	0.001 *	0.001 *
SNPog (^o^)	0.94	1.19	<0.001 *	1.05	2.28	0.020 *	1.08	2.09	0.008 *	0.951
SN/MnPl (^o^)	−0.02	2.10	0.958	0.39	2.58	0.422	−0.10	3.02	0.852	0.503
SN/MxPl (^o^)	−0.44	2.16	0.281	0.10	2.77	0.847	−0.20	1.98	0.578	0.629
Wits (mm)	−5.98	4.52	<0.001 *	−3.07	4.02	0.001 *	0.32	4.04	0.668	0.002 *
Naperp-A (mm)	−0.35	2.53	0.464	−1.80	3.57	0.011 *	0.03	2.84	0.959	0.033 *
Dental										
U1/MxPl (^o^)	−4.17	6.09	0.001 *	−11.99	9.42	<0.001 *	−10.39	10.29	<0.001 *	0.537
L1/MnPl (^o^)	5.98	6.74	<0.001 *	−3.71	6.39	0.004 *	−4.33	8.13	0.007 *	0.744
U1/L1 (^o^)	−2.27	7.55	0.117	15.42	11.96	<0.001 *	14.65	11.71	<0.001 *	0.803
L1/APog (mm)	3.10	2.42	<0.001 *	−0.45	2.28	0.299	−1.42	2.81	0.010 *	0.151
Overjet (mm)	−5.29	3.08	<0.001 *	−4.50	2.36	<0.001 *	−3.51	2.91	<0.001 *	0.158
Overbite (mm)	−2.42	2.16	<0.001 *	−1.59	1.55	<0.001 *	−1.44	1.75	<0.001 *	0.718
Soft tissue										
Facial Convexity (^o^)	−3.24	3.01	0.001 *	−4.55	3.19	0.001 *	−2.32	3.98	0.003 *	0.021 *
Holdaway Angle (^o^)	−3.74	3.93	<0.001 *	−4.29	4.12	<0.001 *	−4.10	3.89	<0.001 *	0.856
Nasolabial angle (^o^)	3.76	8.89	0.031 *	5.06	12.29	0.035 *	2.71	10.79	0.179	0.438
Facial angle (^o^)	1.21	2.81	0.028 *	0.79	3.57	0.244	1.15	2.97	0.042 *	0.672
UL-E (mm)	−1.49	1.69	<0.001 *	−2.40	2.17	<0.001 *	−2.66	2.13	<0.001 *	0.640
UL-S (mm)	−1.50	1.46	<0.001 *	−2.12	1.75	<0.001 *	−2.28	1.86	<0.001 *	0.741
UL-SnPog’ (mm)	−1.26	1.44	<0.001 *	−1.51	1.67	<0.001 *	−1.78	1.86	<0.001 *	0.570
LL-E (mm)	−0.09	1.87	0.798	−2.61	2.24	<0.001 *	−2.95	2.32	<0.001 *	0.569
LL-S (mm)	0.29	2.02	0.446	−2.27	2.10	<0.001 *	−2.75	2.23	<0.001 *	0.397
LL-SnPog’ (mm)	0.44	2.28	0.306	−1.81	2.15	<0.001 *	−2.32	2.30	<0.001 *	0.386

^#^ paired *t*-test; ^##^ independent *t*-test; * *p* < 0.05.

**Table 5:** Intergroup comparison of post-treatment outcomes.

	Two-Phase Group (n = 29)		One-Phase Group (n = 30)		*p* Value ^#^
Variables	Mean	SD	Mean	SD	
Skeletal					
SNA (^o^)	80.95	3.65	80.60	2.54	0.671
SNB (^o^)	76.95	3.46	76.41	2.49	0.628
ANB (^o^)	4.01	1.57	4.19	1.54	0.709
SNPog (^o^)	77.27	3.63	77.28	2.80	0.993
SN/MnPl (^o^)	36.87	5.54	36.76	6.37	0.946
SN/MxPl (^o^)	9.27	2.84	9.93	2.95	0.385
Wits (mm)	−3.74	3.26	−2.46	4.21	0.200
Naperp-A (mm)	−1.87	4.01	−1.45	3.21	0.661
Dental					
U1/MxPl (^o^)	110.71	5.31	111.04	7.37	0.845
L1/MnPl (^o^)	96.74	6.59	94.26	8.14	0.206
U1/L1 (^o^)	124.98	8.19	127.88	9.68	0.220
L1/APog (mm)	3.99	1.83	2.78	2.10	0.022 *
Overjet (mm)	3.17	0.64	3.42	0.85	0.213
Overbite (mm)	1.69	0.87	1.92	0.76	0.290
Soft tissue					
Facial Convexity (^o^)	13.72	4.67	12.74	4.89	0.433
Holdaway Angle (^o^)	15.66	4.14	13.67	4.04	0.068
Nasolabial angle (^o^)	94.07	9.47	94.83	11.59	0.782
Facial angle (^o^)	88.31	3.64	88.56	3.14	0.779
UL-E (mm)	−0.07	1.82	−0.83	2.15	0.152
UL-S (mm)	2.74	1.57	2.30	1.92	0.338
UL-SnPog’ (mm)	6.42	1.69	6.23	1.90	0.680
LL-E (mm)	1.74	1.83	0.79	2.28	0.084
LL-S (mm)	3.02	1.59	2.42	2.16	0.224
LL-SnPog’ (mm)	4.76	1.63	4.45	2.13	0.535

^#^ Independent *t*-test; * *p* < 0.05.

**Table 6:** Classification results of the stepwise discriminant analysis.

	Predicted Group Membership		Total
Original Group Membership	Two-Phase Group	One-Phase Group	
Two-phase group	72.4% (n = 21)	27.6% (n = 8)	29 (100%)
One-phase group	30.0% (n = 9)	70.0% (n = 21)	30 (100%)

## Data Availability

The data presented in this study are available on request from the corresponding author.

## Supplementary Materials

The following supporting information can be downloaded at: https://www.mdpi.com/article/10.3390/jcm11247428/s1, Table S1: Subgroup analysis of the treatment effects between Herbst and Twin-block.
