# Supplementary material for: Effects of Two-Phase Treatment with Functional Appliances Followed by Extraction versus One-Phase Treatment with Extraction in Class II Growing Patients: A Case–Control Study
Source: J Clin Med. 2022 Dec 15;11(24):7428. doi: 10.3390/jcm11247428 (PMC9785998; doi:10.3390/jcm11247428)
Supplement: Supplementary file 1 [file jcm-11-07428-s001.zip › jcm-2044870-supplementary.pdf]

Supplementary Table S1. Subgroup analysis of the treatment effects between Herbst and Twin-block

	Two-phase group (n=29, T1-T3)				
	Herbst subgroup (n=18)		Twin-block subgroup (n=11)		P value#
Variables	Mean	SD	Mean	SD	
Skeletal					
SNA (°)	-1.42	2.34	-1.35	1.92	0.942
SNB (°)	0.31	2.64	1.24	2.18	0.335
ANB (°)	-1.72	1.50	-2.57	0.98	0.149
SNPog (°)	0.71	2.50	1.61	1.84	0.310
SN/MnPl (°)	0.66	2.66	-0.05	2.48	0.478
SN/MxPl (°)	-0.18	2.63	0.55	3.06	0.499
Wits (mm)	-2.26	3.45	-4.39	4.68	0.170
Naperp-A (mm)	-2.14	3.53	-1.25	3.73	0.523
Dental					
U1/MxPl (°)	-11.89	10.34	-12.15	8.16	0.946
L1/MnPl (°)	-3.16	5.99	-4.60	7.21	0.566
U1/L1 (°)	14.23	13.17	17.35	9.97	0.505
L1/APog (mm)	-0.18	2.26	-0.89	2.35	0.424
Overjet (mm)	-3.95	2.57	-5.40	1.72	0.110
Overbite (mm)	-1.32	1.70	-2.04	1.18	0.234
Soft tissue					
Facial Convexity (°)	-4.84	3.35	-4.06	3.01	0.532
Holdaway Angle (°)	-3.46	4.69	-5.66	2.59	0.165
Nasolabial angle (°)	4.62	11.88	5.78	13.49	0.809
Facial angle (°)	0.28	3.90	1.62	2.93	0.338
UL-E (mm)	-2.19	2.42	-2.74	1.74	0.524
UL-S (mm)	-2.01	2.00	-2.30	1.29	0.673
UL-SnPog' (mm)	-1.46	1.91	-1.60	1.26	0.833
LL-E (mm)	-2.22	2.52	-3.25	1.59	0.236
LL-S (mm)	-2.07	2.39	-2.34	1.58	0.756
LL-SnPog' (mm)	-1.61	2.40	-1.96	1.78	0.703

#Independent t-test
